# Basic timekeeping deficit in the Beat-based Form of Congenital Amusia

**DOI:** 10.1038/s41598-020-65034-9

**Published:** 2020-05-20

**Authors:** Pauline Tranchant, Isabelle Peretz

**Affiliations:** 1 International Laboratory for Brain, Music, and Sound Research, Montréal, Québec H3C 3J7 Canada; 2 Department of Psychology, University of Montréal, Québec, H3C 3J7 Canada

**Keywords:** Sensory processing, Human behaviour

## Abstract

Humans have the capacity to match movements’ timing with the beat of music. Yet some individuals show marked difficulties. The causes of these difficulties remain to be determined. Here, we investigate to what extend a beat synchronization deficit can be traced to basic timekeeping abilities. Eight beat-impaired individuals who were unable to successfully synchronize to the beat of music were compared to matched controls in their ability to tap a self-paced regular beat, to tap to a metronome spanning a large range of tempi (225–1709 ms inter-tone onsets), and to maintain the tempi after the sounds had ceased. Whether paced by a metronome or not, beat-impaired individuals showed poorer regularity (higher variability) in tapping, with an inability to synchronize at a fast tempo (225 ms between beats) or to sustain tapping at slow tempi (above 1 sec). Yet, they showed evidence of predictive and flexible processing. We suggest that the beat impairment is due to imprecise internal timekeeping mechanism.

## Introduction

When dancing or listening to music, we typically perceive a “beat” or an underlying metronome-like periodicity. We are extremely precise at synchronizing our movements to this auditory beat, without much awareness and without explicit tutoring. Yet, a few individuals have marked difficulties with synchronizing simple movements, such as bouncing their body, clapping their hands, or tapping their finger, with the musical beat^1–4^. This deficit has been described as “beat-deafness” and to date has been diagnosed using musical stimuli. Here, we examine the possibility that the musical deficit arises from an anomaly in the basic timekeeping mechanisms underlying humans’ exquisite ability to synchronize with external rhythms.

Musical deficits can result from abnormal tuning of the auditory system. The most common form concerns the processing of musical pitch which has been ascribed to a faulty mechanism for detecting fine-grained pitch variations^5^. Music requires the processing of much smaller pitch intervals than speech, which uses coarser pitch variations, at least in non-tonal languages. Indeed, the amusic individuals who suffer from a fine-grained pitch deficit report normal understanding of speech and prosody in everyday life. What characterizes them behaviourally is a difficulty with detecting mistuning in singing, including their own, recognizing a familiar tune without the aid of the lyrics, discriminating melodies varying in pitch, and maintaining melodies in short-term memory^6^. It has been associated with abnormal connectivity between the auditory cortex and inferior frontal cortex, mostly in the right cerebral hemisphere^7^. This pitch disorder is hereditary^8^, and molecular analyses are in progress to identify the responsible genes. In sum, research on the pitch-based form of amusia has uncovered links between acoustical pitch, music, brain networks, and genes. Likewise, impairments in musical beat processing represent a complementary and distinct chance to study the neurobiological foundations of rhythm.

Musical beat finding is a complex task. Although there is a sounded event for each beat in some musical genres, this is not always the case. Moreover, periodicities exist at finer and larger temporal scales than the most salient beat. Thus, perception of a musical beat rests on the interaction between acoustical cues and higher-level cognitive organization. Accordingly, impairments in musical beat processing may occur at any of these levels. Nevertheless, key features of synchronization to music include *timing regularity*, *prediction*, and *tempo flexibility*, and deficits in either of these abilities would be detrimental to successful synchronization.

Humans excel at producing regular motor sequences. By the age of four years, children can tap a sequence with adult-like regularity^9^ in the absence of an external metronome. Yet accurate synchronization to metronome-like sequences appears later, around the age of 8 (see Appendix in^10^). In untrained adults, variability of intervals between taps to a metronome is typically around 4%^11^, which corresponds to 20 ms for a tempo of two beats per second (or an interval of 500 ms). In beat-impaired individuals,variability of inter-tap-intervals has been found to be within the normal range in the absence of a pacing metronome but sometimes abnormally high during synchronization to a metronome^2,12^.

Humans, unlike most other species, also tap in advance of expected metronome sounds by a few tens of milliseconds^11,13^. Taps do not follow the sounds as in classic reaction-time situations, supporting the notion that synchronization relies on the generation of predictions regarding the timing of upcoming beat positions and anticipated planning of motor actions^14^. The deficit studied in beat-impaired adults so far does not seem to arise from faulty anticipation. Evidence of anticipatory behaviour has been reported in two beat-impaired cases, Mathieu and Marjorie^12^. Both cases showed negative asynchronies consistent with anticipatory behaviour. If confirmed in the present group of beat-impaired cases (which does not include Mathieu and Marjorie), a spared ability for predictive synchronization would suggest that the disorder emerges at another level, such as inadequate tempo flexibility.

The tempi to which a neurotypical adult can tap in an anticipatory fashion covers a broad range, from 250 ms to 2000 ms between beats^10,11^. However, intervals of 400 to 1200 ms between auditory sounds give rise to the strongest sense of beat^15^. This optimal range of tempi also reflects the usual tempi of natural motor movements^16–19^. The latter are typically measured by assessing the spontaneous motor tempo (SMT) at which individuals move in the absence of external cues^9,10^. Taps are usually best synchronized at an individual’s SMT^20^. Nevertheless, adult human synchronization excels in its adaptation to a broad range of external tempi, making flexibility in synchronization to an auditory beat a remarkable and quite unique feature in nature^21^.

Tempo flexibility in synchronization has not been studied extensively in beat-impaired individuals. The largest tested range in this population is from 450 to 750 ms beat intervals^2^, which corresponds to optimal tempi for synchronization and is limited with respect to what adults can typically achieve when synchronizing to a metronome (i.e., 250–2000 ms). As posited above, one possible explanation for impairments in beat-finding might be a lack of flexibility, with a narrow adaptation around the individualized spontaneous tempo. Accordingly, beat-impaired individuals should have marked difficulties with synchronizing at slower and faster tempi outside their SMT compared to typical participants. We tested this possibility in the present study.

We assessed individual SMT and tempo flexibility in finger-tapping to a metronome in a new group of eight beat-impaired cases who were characterized by an abnormally poor ability to synchronize their taps to music. Based on previous studies^1–4,12^, we expected both SMT and beat prediction at SMT to be normal and that synchronization would deteriorate abnormally for tempi rolling away from the SMT.

## Results

### Study 1: Spontaneous motor tempo (SMT)

The goal of Study 1 was to assess participants’ SMT for tapping. To this aim, the first three tapping sequences collected at the beginning of each session were analyzed in order to avoid potential carry-over effects of synchronization-continuation tempi on SMT.

**Figure 1:**
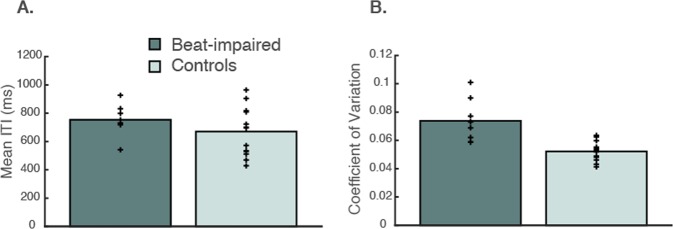
Spontaneous tapping tempo averaged over the six sequences produced before the synchronization-continuation task (**A**) and coefficient of variation (**B**). Each cross represents one individual.

There were large variations in mean spontaneous inter-tap-intervals (ITIs), ranging from 391 ms to 1214 ms, in line with other studies^10^ (Fig. 1A), with no difference between groups, *β* = −84.15, *SE* = 66.23, *t*(20) = −1.42, *p* = 0.22. This broad range of individual SMTs will be taken into consideration when evaluating synchronization abilities in Study 2.

Unexpectedly, the beat-impaired group was found to be less regular in their SMTs than matched controls (Fig. 1B). The coefficient of variation (CV), which is a common measure of temporal variability, corresponds to the standard deviation of the ITIs divided by the mean ITI. CV scores indicated higher variability, hence poorer regularity, in nearly all the beat-impaired cases (*M* = 0.07) compared to the control group (*M* = 0.05), *β* = − 0.022, *SE* = 0.0046, *t*(20) = −4.63, *p* < 0.001.

Irregularity in SMT has not previously been reported in the beat-based form of amusia^1–4,12^. The high variability displayed by beat-impaired individuals in the present study may be related to the larger number of measures as well as the larger number of beat-impaired cases considered here. Nevertheless, to allow for comparison, we applied the procedure used by Sowinski & Dalla-Bella^2^ and examined the SMT showing the lowest CV (highest regularity) from the first two sequences produced by each participant. This analysis revealed no statistically significant difference between groups, *t*(14) = −1.75, *p* = 0.10 (Welch’s two-sample t-test), in line with findings found in^2^. Therefore, the first analysis, which included more data (186 taps per participant) than Sowinski & Dalla Bella (53 taps per participant on average), appears more sensitive to the presence of a deficit. Note that the CV remained higher in the beat-impaired group (*M* = 0.09) than in the control group (*M* = 0.06) when considering the averaged CV over the other 14 SMT sequences measured over the two sessions, *β* = − 0.030, *SE* = 0.006, *t*(20) = −5.03, *p* < 0.001.

Of note, regularity in spontaneous tapping was unrelated to individual tempo. There were no significant correlations between CV and SMT; *r*(20) = 0.26, *p* = 0.23 across all participants and *r*(6) = 0.28, *p* = 0.50 for the beat-impaired group only.

### Study 2: Flexibility

The temporal irregularities noted in spontaneous tapping in beat-impaired individuals as compared to controls suggest that the capacity to maintain regularity is subnormal in the beat-based form of congenital amusia. Providing an external aid via a metronome might bring performance to normal. To be effective as an aid, it is likely that the metronome rate should be proximal to the individual SMT. Furthermore, continuation (without the external aid) is expected to be disrupted, especially for stimulus rates distant from the SMT^20^. The goal of Study 2 was to examine these predictions.

In order to test synchronization-continuation at the individual’s optimal tempo, the metronome sequences were matched to each participant’s SMT value obtained in Study 1 for the sequence with the highest regularity (lowest CV over the first three spontaneous sequences measured in each session, as in^2^). Two sequences were used for each condition (SMT, SMT + 50 ms, SMT-50ms; one sequence per session). Synchronisation performance was measured with both the CV to measure the effect of the presence of an external aid relative to spontaneous tapping (Study 1) and circular statistics to measure synchronization consistency.

**Figure 2:**
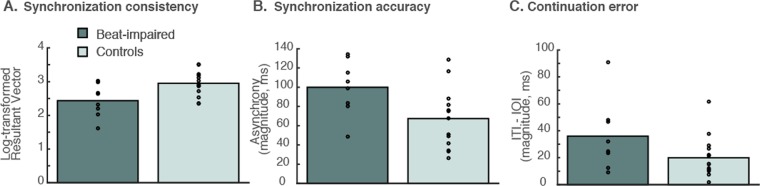
Synchronization-continuation at participants’ spontaneous tempo (SMT) for beat-impaired and control participants. The average score for each participant is represented by a dot. Bar graphs represent arithmetic means. For synchronization consistency (**A**), the higher the score the better the performance; for synchronization accuracy (**B**) and continuation error (**C**), the lower the score the better the performance.

The CVs of ITIs for inter-onset-intervals (IOIs) set to the individual SMT and SMT ± 50 ms were again found to be higher in the beat-impaired (*M* = 0.07) than in the control group (*M* = 0.05), *β* = −0.016, *SE* = 0.0031, *t*(20) = −5.15, *p* < 0.001. Thus, tapping at one’s optimal tempo with an external beat is not sufficient to bring beat-impaired performance into the normal range. However, the CV does not capture synchronization consistency because it does not take the stimulus into account. For example, a sequence of taps could be highly regular (low CV) but not aligned with the tones. Thus, the log transformed resultant vector (see Data Analysis in Methods) was considered here. This measure confirms lower consistency in the beat-impaired group than in the control group, *β* = 0.70, *SE* = 0.19, *t*(20) = 3.77, *p* = 0.0012 (Fig. 2A).

Anticipation of the metronome tones, by tapping before the tone onsets, was present in both groups. The asynchronies between taps and tones were computed by subtracting the closest tone onset time from the tap time. The taps were further ahead of the tones in the beat-impaired group (*M* = −103 ms) than in the control group (*M* = −56 ms), *β* = −48, *SE* = 11, *t*(20) = −4.15, *p* < 0.001 (Fig. 2B).

Continuation performance was similar to synchronization, with lower regularity (higher CV) in the beat-impaired (*M* = 0.069) than in the control group (*M* = 0.056), *β* = −0.013, *SE* = 0.0045, *t*(20) = −3.01, *p* = 0.0069. The ability to maintain the tempo of the metronome, as measured by the distance between the mean ITI and the stimulus IOI (referred to as continuation error in Fig. 2C), was also poorer in the beat-impaired group, *β* = −17, *SE* = 7, *t*(20) = −2.72, *p* = 0.035.

To summarize, the results showed that even at each participant’s most comfortable tempo and with external isochronous tones, tapping regularity generally remained poorer in the beat-impaired group than in the control group. Synchronization consistency, anticipation of tones, and continuation were also slightly poorer in the beat-impaired group despite large scores overlap between groups. In what follows, we assessed to what extent less comfortable stimulus tempi disrupted performance.

**Figure 3:**
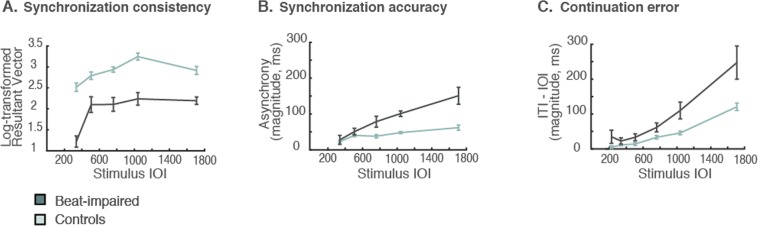
Mean synchronization and continuation scores at 337, 506, 759, 1139, 1709 ms IOIs (and at 225 ms for continuation). The graphs show arithmetic means and standard error bars corrected for repeated measures. For 337 ms, only the sequences with significant Rayleigh tests are included in the calculation of synchronization consistency and accuracy.

When tempi were imposed with beat intervals between 225 and 1709 ms, beat-impaired performance in both synchronization and continuation was poor, particularly for the fastest tempo (IOI = 225 ms; not displayed in Fig. 3). The Rayleigh test revealed a failure to period-match (*p* > 0.05) the fastest IOI in six of the eight beat-impaired cases whereas all of the participants from the control group were able to period-match. Note that this difficulty does not seem to arise from biomechanical or motor limitations, because beat-impaired participants were generally producing fast rates when tapping to fast stimuli (Table 1). Because sequences were unsuccessfully period-matched with the fastest stimulus rate in most beat-impaired individuals, this tempo was not included in subsequent analyses of synchronization consistency and accuracy. Synchronization difficulties were also observed for the second fastest rate (337 ms); a failure to period-match at that tempo was observed in three (out of 16) sequences from three beat-impaired cases, while all but one sequence were period-matched in controls. These failed sequences were not included in subsequent analyses of synchronization consistency and accuracy.

**Table 1:** Mean ITI when tapping to the fastest stimulus (IOI = 225 ms) in beat-impaired individuals and the control group.

	ITI (ms)
Beat-impaired participants	
1	206
**2**	**233**
3	254
4	301
5	199
6	233
**7**	**231**
8	210
Control participants	
*M* (range)	225 (223–227)

The two beat-impaired individuals with ITIs consistent with the stimulus IOI (as indicated by the Rayleigh test) are in bold.

Even at slower tempi, synchronization consistency as measured by the log-transformed resultant vector (Fig. 3A) remained lower in the beat-impaired group compared to the control group, *β* = 0.90, *SE* = 0.14, *t*(20) = 6.52, *p*  < 0.001. In both groups, performance decreased at slower tempi, with an effect of Stimulus Tempo, *β* = 0.19, *SE* = 0.040, *t*(192) = 4.67, *p* < 0.001. There was no interaction with Group, *β* = −0.089, *SE* = 0.080, *t*(192) = −1.11, *p* = 0.27. Post-hoc comparisons for the effect of Stimulus Rate (with Bonferroni-Holm *p*-value adjustment) showed lower consistency for 337 ms compared to 759, 1139 and 1709 ms (all *p*-values < 0.001) and for 506 ms compared to 1139 ms (*p* = 0.015) across both groups. The correlation between synchronization consistency scores at SMT (and SMT ± 50 ms) and synchronization consistency scores at tempi in the range 337–1709 ms (removing scores for imposed tempi lying within ± 50 ms of an individual’s SMT) reached significance in the beat-impaired group, *r*_6_ = 0.87, *p* = 0.0055 (*r*_12_ = 0.44, *p* = 0.11 in the control group). This result points towards a single origin of impairment in the reduced capacity to consistently match metronome tones, irrespective of its rate.

Anticipation of metronome tones was again observed in both groups: mean asynchronies were negative for 84% and 85% of the sequences in the beat-impaired and control group, respectively. Taps preceded tone onsets in both groups, but to an anomalously greater degree in the beat-impaired group for the slow metronome tempi, as indicated by an interaction between Group and Stimulus Tempo, *β* = −29, *SE* = 5.31, *t*(192) = −5.54, *p* < 0.001 (Fig. 3B). The larger asynchronies for slower rates indicate a tendency for the beat-impaired group to underestimate the stimulus IOI.

In continuation (Fig. 3C), the error tended to increase with slower tempi in both groups. However, this tendency was larger in the beat-impaired than in the control group, as supported by an interaction between Group and Stimulus Tempo, *β* = −42, *SE* = 10, *t*(216) = −4.36, *p* < 0.001. Groups did not differ for the fast and moderate tempi (225, 337, 506 and 759 ms; all *p*-values > 0.05) but did for the slower tempi (1139 ms, *p* = 0.0028; 1709 ms, *p* < 0.001). Thus, beat-impaired participants were as able as controls to maintain fast and moderate tempi after the stimulus had stopped. This suggests that the imposed tempi had an outlasting effect on all participants’ performance, including the beat-impaired ones.

**Figure 4:**
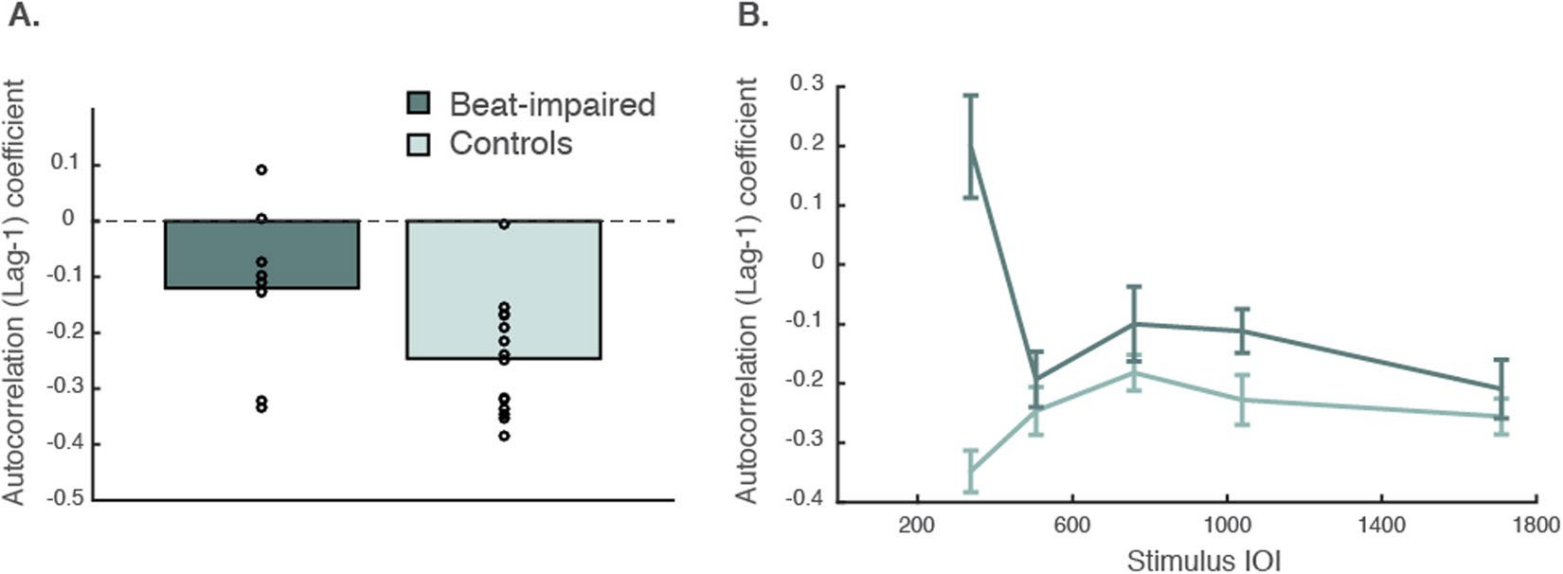
Autocorrelation coefficient at SMT (**A**) and imposed rates (**B**) for the beat-impaired and control group. Each dot represents a participant in A. The error bars in B represent standard errors of the mean, adjusted for repeated measures.

We examined error correction during tapping with a metronome, as done with beat-impaired cases in a previous study^2^. At comfortable tempi (SMT; Fig. 4A), we found a negative lag-1 autocorrelation coefficient in both beat-impaired (M = −0.12) and control (M = −0.25) groups, which is typical of effective correction in adults^22^. This finding, added to the overlap between beat-impaired and control group scores at SMT (see Fig. 2), suggests a window for improved beat processing around one’s comfortable tapping rate. At fixed tempi, there was an interaction between Stimulus Tempo and Group, *β* = 0.10, *SE* = 0.032, *t*(192) = 3.28, *p* = 0.0012. This interaction was due to the observation of a positive lag-1 autocorrelation coefficient in the beat-impaired group at 337 ms (Fig. 4B), while values remained negative for all other tempi in both groups. Thus, the beat-impaired group showed evidence of error correction except for the fastest tempo. Lag-one autocorrelation during synchronization was most negative in the beat-impaired group at the slowest tempo (1709ms), yet not significantly different from the other tempi except at the fastest one (all p > 0.05; bonferroni-holm correction for multiple comparisons). This is consistent with slightly higher synchronization consistency found at slower tempi as compared to other tempi in the beat-impaired group. Large systematic negative asynchronies are compatible with higher consistency, since the latter is a measure of period-matching (for further explanation see ^2^). Beat-impaired participants tapped at the correct period, but ahead of the metronome tones. A possible explanation for preserved error correction at slow tempo is that the undershoot-overshoot pattern typical of a negative lag-1 autocorrelation is centered around an internal beat that is systematically ahead of the tones.

As a control measure, all of the analyses presented above were also performed with groups of equal sizes, by considering 8 control participants instead of 14, and the results remained the same.

**Figure 5:**
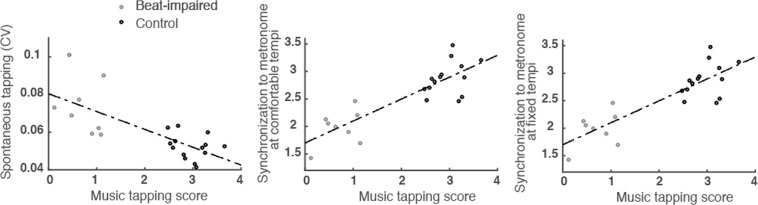
Regularity in spontaneous tapping (CV), synchronization consistency to metronome (transformed circular variance) at comfortable tempo, and synchronization consistency to metronome at fixed tempi (337–1709 ms) are presented as functions of individual scores in tapping to music.

We also measured correlations between performance on the musical stimuli (the BAT screening test) and performance with or without a metronome. No significant correlations were found within each group, likely due to their limited size. When considering all participants, significant correlations emerged between the music (BAT) tapping score and (1) CV (regularity) in spontaneous tapping (SMT), *r* = 0.72, *p* < 0.001; (2) synchronization consistency at comfortable tempo, *r* = 0.53, *p* = 0.012; and (3) synchronization consistency at fixed tempi (337–1709 ms), *r* = 0.84, *p* < 0.001 (Fig. 5). These findings provide support to the notion that tapping to music is related to tapping regularity in general, whether there is an external metronome or not.

Finally, in order to determine potential sources of variance in the timing of taps, we applied the model of Wing & Kristofferson to our data^22,23^.The model states that the total ITI variance in the continuation part of synchronization-continuation sequences can be decomposed into “timekeeper” and “motor implementation” variance. However, the model could not be applied to most data because at the slowest tempi (1139 and 1709ms), more than 50% of the trials did not meet the model assumption of lag-one auto-correlation coefficient being between 0 and −0.5. Similarly, at the fastest tempi (225 and 337 ms), most beat impaired participants were unable to perform the synchronization task. For the remaining 509 ms and 739 ms tempi, 18% of trials were excluded in the beat-impaired group and 32% of trials were excluded in the control group because they did not meet the model’s assumption of lag-one auto-correlation coefficient being between 0 and −0.5. For the remaining trials higher variance was observed in the beat-impaired group for both timekeeper, *t*(20) = 2.49, *p* = 0.021, and motor, *t*(24) = −3.39, *p* = 0.0024 implementation variances. These results should be considered with caution given the limited number of data considered. Yet, they do not suggest a distinctive contribution of an internal time-keeping over a motor implementation problem in our sample of beat-impaired individuals.

## Discussion

We show that individuals initially identified with a deficit in musical beat processing have problems with basic timekeeping. Beat-impaired participants showed poorer regularity (higher variability) than neurotypical nonmusicians when tapping, whether paced by a metronome or not. They also showed an inability to synchronize at a fast tempo (225 ms between beats) and to sustain tapping at slow tempi (above 1 sec). Yet, they can tap at very fast and very slow speed, suggesting that they do not have biomechanical limitations. Morever, the beat-impaired individuals show evidence of predictive processing, by anticipating isochronous beats, and of error correction when tracking the metronome. However, none of these markers of beat processing were as precise as those obtained in typical performers. We discuss below to what extent imprecision in basic timekeeping might explain their musical deficit.

The observation of a systematic higher variability across conditions in individuals who have problems to track the musical beat as compared to matched controls points to imprecision at the level of a timekeeping mechanism that does not appear related to a motor limitation. The spontaneous tapping tempo of the beat-impaired participants is not different from the tempo of neurotypical controls. What differs is the regularity of their tapping. Irregularity in spontaneous tapping was not expected, as it has not been reported previously^2,4,12^ except in our own recent work^24^. The reason might come from the fact that our study includes one of the largest samples of individuals with a beat-finding disorder and collected over twice as many tapping data. Variability in timekeeping is not restricted to auditory-motor synchronization since it affected tapping both in the presence of auditory feedback from a metronome and in its absence during spontaneous tapping. Beat-impaired individuals also display more difficulty with adapting their tapping to temporally changing signals, such as phase and period perturbations in a metronome sequence^12^. Finally, they have problems to track the beat when judging if a surimposed metronome matches the beat, while no motor response is required. These results point to a lack of precision in internal timekeeping mechanism, sometimes called “intrinsic rhythmicity”^24,25^.

Yet, the deficit is subtle. As mentioned, the beat-impaired individuals show evidence of predictive processing by anticipating isochronous beats. They can synchronize to a relatively large range of metronome tempi, thereby showing some flexibility. In addition, they can sustain the same tempo after a metronome has stopped, but with abnormally large synchronization asynchronies and continuation deviations at slow tempi. The fact that asynchronies and deviations from stimulus tempo are larger for slow than for fast tempi may reflect that long intervals (slow tempo) leave more room to deviate than short intervals (fast tempo). Together with the breakdown of synchronization with very fast tempi (225 ms between tones) and poor error-correction at moderately fast tempi (337 ms between tones), our results suggest that the hallmark of the impaired intrinsic rhythmicity of beat-impaired individuals lies in an alteration of the fine-tuning that characterizes typical human adults’ tapping. The deficit is not specific to music but extends to other auditory rhythms, provided by a metronome as here, or in speech and song as in^24^. Thus, the results point to the presence of a basic deficiency in timekeeping mechanisms that affect entrainment in general.

The reason why the deficit is best identified in tasks requiring tapping (or bouncing or clapping^1,3^) with music is probably related to the nature of the task. Moving to the beat is spontaneous and widespread while metronome tracking or perceptual judgments, such as those used in the Montreal Battery for the evaluation of amusia (MBEA), are rarely performed in everyday life. Moreover, the meter test of the MBEA lacks sensitivity due to the high variability of typical scores, with a cut-off score of 2 SD below the typical mean lying at chance level.

The key question is how to best account for the observed imprecision of internal rhythmicity in congenital amusia. The model we proposed in^24^ is that the imprecision in regular tapping accompanied with normal but imprecise sensitivity to external rhythm, may be caused by broader tuning of self-sustained neural oscillations in the beat-impaired brain. An idea that is currently gaining increasing strength is that auditory-motor synchronization capitalizes on the tempi of the naturally occurring oscillatory brain dynamics, such that moments of heightened excitability (corresponding to particular oscillatory phases) become aligned to the timing of relevant external events (for a recent review, see^26^). In the beat-impaired brain, the alignment of the internal neural oscillations to the external auditory beats would take place, as shown by their sensitivity to a broad range of metronome tempi, but it would not be sufficiently well calibrated to allow precise entrainment. This account is currently under investigation in our laboratory.

To conclude, we show that deficient synchronization to music can be traced to an imprecise core timekeeping mechanism. The hypothesised operation of more broadly-tuned brain oscillators in beat-impaired individuals provides a useful framework to understand poor performance in tracking the musical beat in particular and putatively to understand the exquisite human ability to tune to auditory rhythms in general.

## Methods

### Participants

Eight beat-impaired participants (6 females; mean age: 27.1 years, *SD* = 2.2) were matched for years of education and years of music and dance training to 14 control participants (9 females; mean age: 26.6 years, *SD* = 2.3). All participants were university students or recent graduates, and none had history of neurological or motor disorders. All beat-impaired cases were selected on the basis of poor synchronization to music. The beat processing disorder was assessed with a customized version of the Beat Alignment Test (BAT)^27^, in which participants tapped to the beat of 10 songs of different genres, each presented twice.

**Figure 6:**
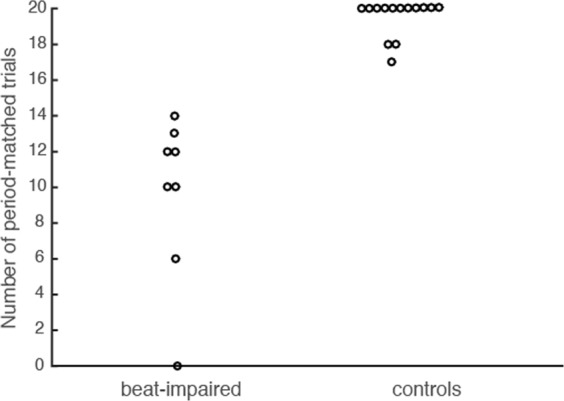
Number of period-matched trials out of 20 per participant in each group. A dot represents one participant.

In order to assess whether taps were consistent with the beat period of each song, circular statistics^28^ and Rayleigh tests were used, after transforming taps into vectors on a circle (one vector for each tap; see Data Analysis section below for other details). A significant Rayleigh test (*p* < 0.05) indicates success in period matching, indicating that inter-tap intervals (ITIs) are consistent with the stimulus inter-onset-interval (IOI). As evident in Fig. 6, the beat impairment affected period matching; there was no overlap between the number of trials that were period-matched with the beat of the songs between the beat-impaired and control participants. To confirm the presence of a beat synchronization deficit, the resultant vector of taps was used to compare the performance of the beat-impaired cases to normative scores obtained from 41 typical synchronizers (23 females, mean age: 26.6 years, *SD* = 4.4;^25^). The resultant vector is the circular mean of the vectors computed for each stimulus; it is a measure of consistency between ITIs and IOIs. It is bounded by zero and one; a value close to zero indicates a random distribution of vectors around the circle, hence low consistency between ITIs and IOIs, while a score close to one indicates high consistency between ITIs and IOIs. In the current study, this measure was negatively skewed and was transformed using a log function. For each participant, the log-transformed values were averaged across the 20 trials, providing an index score of individual performance. The beat-impaired individuals’ values (mean and range) were two *SDs* below the mean of the typical group (cut-off = 1.74; mean = 2.71, SD = 0.48), which confirmed their poor beat-processing abilities with music.

All participants were screened for the presence of musical deficits other than beat synchronization. The beat-impaired cases were evaluated with the Montreal Battery of Evaluation of Amusia (MBEA^29^), which assesses melodic pitch, rhythm, and meter processing in music, as well as musical memory. The melodic composite score is averaged here over three tests (scale, contour and interval), each comprising 30 trials, assessing pitch processing in a melodic context. The rhythm test evaluates a participant’s ability to judge whether two short melodies are different with regard to the duration of two adjacent intervals (while maintaining the beat structure). The meter test assesses a participant’s ability to judge whether short melodies have an underlying pattern of strong and weak beats that corresponds to either a march (ONE two ONE two) or a waltz (ONE two three ONE two three). According to recent norms^30^, participants in the current study obtained normal melodic composite and rhythm scores (Table 2). Only two out of the eight cases scored below cut-off on the meter test (Table 2); however, note that this test may lack sensitivity, with a cut-off score close to chance (17/30 compared to 15/30). Beat perception abilities were further evaluated with the perception part of the BAT^27^. Participants are presented with the same songs as used in the BAT tapping task used to screen the beat-impaired cases. Here, no tapping response is required. Instead, participants judge whether a superposed metronome is aligned or not with the beat. All participants were tested twice on different days, with several months between the two sessions, and the average score was computed. Using the d’ sensitivity index we found poorer scores in beat-impaired compared to control participants, *t*(20) = 5.35, *p* < 0.001. Thus, the beat-impaired group shows a deficit in both beat perception and synchronization.

**Table 2:** Individual scores of the beat-impaired cases on the melodic (scale, contour, interval), rhythm, and meter tests of the Montreal Battery of Evaluation of Amusia.

Beat-impaired participant	Melodic Composite Score (/30, cut-off = 21.4)	Rhythm (/30, cut-off = 22),	Meter (/30, cut-off = 17)
1	26.0	22	25
2	24.7	26	24
3	26.3	28	20
4	25.0	26	19
5	24.3	25	21
6	23.7	27	**13**
7	23.0	26	**16**
8	25.3	25	23

Scores below cut-off (2 SDs below the mean^30^) are in bold.

The musical abilities of the matched controls were tested with the Online Test of Amusia, for which they all obtained normal scores^31^. Finally, all participants performed within the normal range for tests of verbal working memory (Digit Span) and non-verbal reasoning (Progressive Matrices) from the WAIS-III (Wechsler Adult Intelligence Scale, 3^rd^ edition^32^).

All participants gave written informed consent in accordance with the Declaration of Helsinki and received monetary compensation for their participation. All procedures were approved by the Research Ethics Council for the Faculty of Arts and Sciences at the Université de Montréal.

### Material and procedure

**Figure 7:**
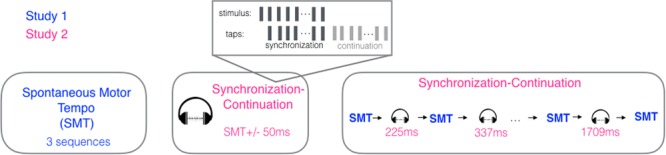
Schematic of the experimental procedure. Participants started each session by tapping 31 taps three times, in order to measure Spontaneous Motor Tempo (SMT; Study 1). They proceeded to tap at their SMT but this time with an auditory metronome, continuing to tap after the metronome ceased until asked to stop. Next, they tapped at the imposed variable metronome tempi (Study 2) interspersed with SMT trials. The whole procedure was repeated in a second session.

The design of the experiment is schematized in Fig. 7. Each participant started each session by tapping in silence (no stimulus) for three trials of 31 taps at their most comfortable rate, in a constant and regular fashion, to measure their SMT. Next, each participant was invited to tap with a metronome (synchronization) and to continue and maintain the tempo after the sounds had stopped (continuation). Before each trial, a participant was primed with a short version of the stimulus (10 tones) and instructed to listen without moving. Then, the participant was invited to tap in synchrony with the metronome, starting as soon as they felt comfortable, for 31 isochronous tones (440 Hz, 200 ms duration), with the stimulus’ IOI set to their SMT as computed from the previous spontaneous tapping trials. They also tapped to stimuli corresponding to their SMT plus or minus 50 ms (SMT + /−50). The order of SMT + 50 and SMT-50 was counter-balanced between participants and inverted in a second testing session. An auditory beep indicated when to start and when to stop, that is, after 31 taps were made.

The session ended with six synchronization-continuation trials of varying IOIs interleaved with seven spontaneous tapping sequences (Fig. 7). Stimuli IOIs were fixed and consisted of 225, 337, 506, 759, 1139, and 1709 ms, as used in^10^. As previously, a short version of a given stimulus (10 tones) was presented before each synchronization-continuation trial for preparation. The synchronization-continuation trials were presented in a descending (fast to slow) or ascending (slow to fast) order, which was counter-balanced between participants and inverted in the second session. Each participant was tested in all conditions twice over two separate sessions, with a minimum of six days between sessions. Each session lasted approximately 30 minutes.

Taps were made on a square force-sensitive resistor (3.81 cm, Interlink FSR 406) placed on a table in front of the participant. The resistor was connected to an Arduino Duemilanove transmitting timing information to a PC (HP ProDesk 600 G1, Windows 7) via the serial USB port. Tap times were recorded and stimuli were generated with a customized program in MAX/MSP (Cycling’ 74) and presented at a comfortable level through headphones (DT 770 PRO, Beyerdynamics).

### Data analysis

For each participant, there were 20 spontaneous tapping sequences and 18 synchronization-continuation sequences, each consisting of 31 taps. The data were analyzed in Matlab (R2014b, Mathworks) with linear and circular methods, using the CircStat Toolbox^33^. In Study 2, the difference between the time of a tap and the time of the corresponding metronome tone onset (i.e. the asynchrony) was transformed into the relative phase on a unit circle, with 0 indicating perfect alignment between taps and tones, and with negative and positive values indicating taps preceding or following the tones, respectively. Whether a sequence of taps (trial) was successfully synchronized with the metronome was assessed using the Rayleigh test of uniform distribution of the relative phases on the unit circle. A significant Rayleigh test (*p* < 0.05) indicated that ITIs were consistent with the metronome’s period. The resultant vector is the circular mean of the vectors, computed for each trial. As the resultant vector was transformed using a log function (new score = − log (old score)), a higher score indicates higher consistency. Note that the resultant vector was only analyzed when the corresponding Rayleigh test was significant.

For each metronome sequence, mean asynchrony, and lag-1 autocorrelations were as well calculated for individual trials. The mean asynchrony was used as an index of synchronization accuracy. In addition, the relationship between adjacent ITIs was assessed using the lag-1 autocorrelation of the ITIs (e.g. ^34,35^,). A positive lag-1 autocorrelation would be characterized by successive short or successive long tap intervals and would suggest that tap intervals were drifting away from the IOI of the metronome, while a negative lag-1 autocorrelation would be characterized by alternations between short and long tap intervals, which would suggest that error correction mechanisms were preventing the drift (e.g. ^36^,).

Statistical analyses were performed with linear mixed-effects models, using the “lme4” package^37^ in R (R Core Team, 2015). The models included a random term for Participant to account for repeated measures. Factor IOI was centered and scaled using the *scale* function in R before running mixed-effects statistical models, so that factors Group and IOI were on comparable scales. We used mixed-effects models rather than ANOVA because they do not require prior averaging of the data and have more statistical power in the case of unbalanced designs such as different group sizes. Corresponding *p*-values and degrees of freedom were computed from the Satterthwaite approximations, using the “lmerTest” package^38^ in R. For post-hoc analyses, pairwise comparisons were performed with the “lsmeans” package^39^ in R, which computes least-square rather than arithmetic means to account for unbalanced designs. Because no statistical differences were found between the data collected in session one or session two (all *p* > 0.15), these data were combined in subsequent analyses.

## Data Availability

The datasets generated during and/or analysed during the current study are available from the corresponding author on reasonable request.
